# Sugar transport for enhanced xylose utilization in *Ashbya gossypii*

**DOI:** 10.1007/s10295-020-02320-5

**Published:** 2020-10-09

**Authors:** David Díaz-Fernández, Gloria Muñoz-Fernández, Victoria Isabel Martín, José Luis Revuelta, Alberto Jiménez

**Affiliations:** Metabolic Engineering Group, Departamento de Microbiología Y Genética, Universidad de Salamanca, Campus Miguel de Unamuno, 37007 Salamanca, Spain

**Keywords:** Sugar transport, Glucose, Xylose, *Ashbya gossypii*, CRISPR/Cas9

## Abstract

The co-utilization of mixed (pentose/hexose) sugars constitutes a challenge for microbial fermentations. The fungus *Ashbya gossypii*, which is currently exploited for the industrial production of riboflavin, has been presented as an efficient biocatalyst for the production of biolipids using xylose-rich substrates. However, the utilization of xylose in *A. gossypii* is hindered by hexose sugars. Three *A. gossypii* homologs (*AFL204C, AFL205C* and *AFL207C*) of the yeast *HXT* genes that code for hexose transporters have been identified and characterized by gene-targeting approaches. Significant differences in the expression profile of the *HXT* homologs were found in response to different concentrations of sugars. More importantly, an amino acid replacement (N355V) in AFL205Cp, introduced by CRISPR/Cas9-mediated genomic edition, notably enhanced the utilization of xylose in the presence of glucose. Hence, the introduction of the *afl205c-N355V* allele in engineered strains of *A. gossypii* will further benefit the utilization of mixed sugars in this fungus.

**Electronic supplementary material:**

The online version of this article (10.1007/s10295-020-02320-5) contains supplementary material, which is available to authorized users.

## Introduction

*Ashbya gossypii* is a filamentous hemiascomycete that is currently used for the industrial production of riboflavin [1]. Furthermore, the ability of *A. gossypii* to grow using industrial by-products highlights the importance of this fungus as a microbial factory [2, 3].

Engineered strains of *A. gossypii* were described to use xylose for the production of biolipids [4]. However, the utilization of xylose in those strains is hampered by the presence of hexose sugars [3], due to mechanisms affecting the simultaneous utilization of mixed sugars (i.e., glucose and xylose).

Most organisms metabolize mixed sugars sequentially (diauxic growth) and show preference for glucose. This process is regulated by a mechanism called carbon catabolite repression (CCR), which is controlled by the Snf1/Mig1 regulators in *S. cerevisiae* [5], and is further sustained by the absence of high-affinity transporters for pentose sugars [6]. Hence, in *Saccharomyces cerevisiae*, which is evolutionary closely related to *A. gossypii*, the uptake of pentose sugars is facilitated by hexose transporters (Hxt) that are a bottleneck for xylose utilization [7, 8].

Different strategies have been implemented to improve the uptake of pentose sugars including the overexpression of native and engineered *HXT* genes, the overexpression of heterologous xylose transporters and the evolutionary engineering of xylose-utilizing strains [6, 8–12]. For example, in *S. cerevisiae* the overexpression of hexose transporters (Hxt7 and Gal2) resulted in improved pentose consumption in the presence of glucose [13]. In addition, directed evolution of hexose transporters has led to the identification of residues that are important for sugar specificity such as N376 of Gal2 in *S. cerevisiae* [14]. Mutations in the homologous N376 arginine residue of different transporters have been described to improve xylose consumption [14–16]. Also, some native low-affinity transport systems for pentose sugars have been described in xylose-utilizing microorganisms such as *Candida intermedia* and *Scheffersomyces stipitis* [6, 17].

In this work, we sought to investigate the contribution to the xylose uptake of *A. gossypii* homologs of the *HXT1*, *HXT3-7* genes. Gene expression analyses and gene-targeting approaches of the predicted hexose transporters in *A. gossypii* showed that Afl205Cp may have a prominent role in the xylose uptake. Furthermore, the use of the CRISPR/Cas9 genomic editing tool allowed us to engineer a specific mutation (*AFL205C*-N355V) that significantly improved the consumption of xylose in *A. gossypii*.

## Materials and methods

### A. gossypii strains and growth conditions

The *A. gossypii* ATCC 10,895 strain was used and considered the wild-type strain. Other *A. gossypii* strains used in the work are listed in Table 1. *A. gossypii* cultures were initiated with spores (10^6^ spores per liter) and carried out at 28 ºC in MA2-rich medium [18] using either glucose and/or xylose as carbon sources (2% w/v). *A. gossypii* transformation, its sporulation conditions and spore isolation were as described previously [18]. Concentrations of 250 mg/L for geneticin (G418) (Gibco-BRL) were used where indicated.

**Table 1:** *A. gossypii* strains used in this study

*Strain*	*Genotype*	*Phenotype*	*Source*
WT	Wild type (ATCC 10,895)	WT	Our lab stock
*A665 (GXX)*	*P*_*GPD1*_*-GRE3, P*_*GPD1*_*-XKS1, P*_*GPD1*_*-XYL2 (GXX strain)*	xyl^+^^a^	Our lab stock
*A741*	*GXX, afl205cΔ*	xyl^+^	This work
*A742*	*GXX, afl204cΔ*	xyl^+^	This work
*A743*	*GXX, afl207cΔ*	G418^R^, xyl^+^	This work
*A745*	*GXX, afl205cΔ, afl207cΔ*	G418^R^, xyl^+^	This work
*A746*	*GXX, afl204cΔ, afl205cΔ*	G418^R^, xyl^+^	This work
*A747*	*GXX, afl204cΔ, afl207cΔ*	G418^R^, xyl^+^	This work
*A764*	*GXX, afl204cΔ, afl205cΔ, afl207cΔ*	xyl^+^	This work
*A844*	*GXX, P*_*GP1D*_-afl205c*(N355V), P_*GPD1*_*-AFL207C, P*_*GPD1*_*-AFL204C*	xyl^+^	This work
*A847*	*GXX, P*_*GPD1*_*-AFL205C, P*_*GPD1*_*-AFL204C, P*_*GPD1*_*-AFL207C*	xyl^+^	This work

^a^xyl^+^ indicates the ability to grow using xylose as the only carbon source

### Gene manipulation

Gene deletion was carried out using a gene-replacement cassette that was constructed for each gene by PCR amplification of a *loxP-KanMX-loxP* selection marker with the corresponding recombinogenic flanks (see primers in Table S1). Gene overexpression was carried out using the sequence of the constitutive strong promoter of the *AgGPD1* gene that was integrated upstream of the ATG initiator codon of each gene. Overexpression cassettes were PCR amplified using specific primers (Table S1) and comprised a *loxP-KanMX-loxP* selection marker, the *P*_*GPD1*_ sequence and recombinogenic flanks for each gene. The *loxP* repeated inverted sequences present in the *loxP-KanMX-loxP* marker enabled the selection marker to be eliminated and subsequently reused by expressing a Cre recombinase, as described elsewhere [19].

Either a gene-replacement cassette or an overexpression module was used to transform spores of *A. gossypii*. Primary heterokaryotic clones were selected in G418-containing medium, and homokaryon clones were obtained by sporulation of the primary transformants. The correct genomic integration of each deletion/overexpression cassette was validated by analytical PCR followed by DNA sequencing (data not shown). Gene overexpression was further confirmed by qRT-PCR analysis (data not shown).

### Quantitative real-time PCR

Quantitative real-time PCR (qRT-PCR) was carried out as described previously [20]. Primer sequences used for qPCR are listed in Table S1. All real-time PCRs were performed in duplicate and in at least two independent experiments. Relative quantification analyses were carried out using the LightCycler 480 software. The mRNA level of the target genes was normalized using the housekeeping *AgUBC6* gene as a reference.

### HPLC analysis of metabolites

Glucose and xylose from culture media were analyzed with a Waters Alliance 2795 High-performance liquid chromatography system equipped with a REZEX ROA Organic Acid H + (8%) column (25 cm long, 4.6 mm internal diameter) coupled to RI detector (Waters 410). The mobile phase was 0.005 N H_2_SO_4_, and the flow rate was 0.6 mL/seg at 50 ºC of temperature. All samples from culture supernantants were filtered through 0.45-µm filters, and 25 µL of each sample was used for the analyses.

### CRISPR/Cas9 genomic edition of AFL205C

A CRISPR/Cas9 method adapted for *A. gossypii* was used for genomic edition of the *AFL205C* gene. The synthetic guide sgRNA-dDNA comprised both the guide RNA (gRNA) for *AFL205C* targeting and the donor DNA (dDNA) for DSB repair with the mutant sequence containing two point mutations. The CRISPR/Cas9*-AFL205C-N355V* plasmid was assembled as previously described [21].

Spores of *A. gossypii* were transformed with the CRISPR/Cas9*-AFL205C-N355V* plasmid. Positive clones were selected and grown in G418-MA2 media during 2 days to facilitate the occurrence of the genomic edition. Next, loss of the CRISPR/Cas9*-AFL205C-N355V* plasmid was achieved by sporulation of the primary heterokaryotic cloned in SPA media lacking G418. The presence of the CRISPR/Cas9 mutations was verified by analytical PCR and DNA sequencing.

## Results and discussion

### Characterization of *HXT* homologs in *A. gossypii*

**Fig. 1:**
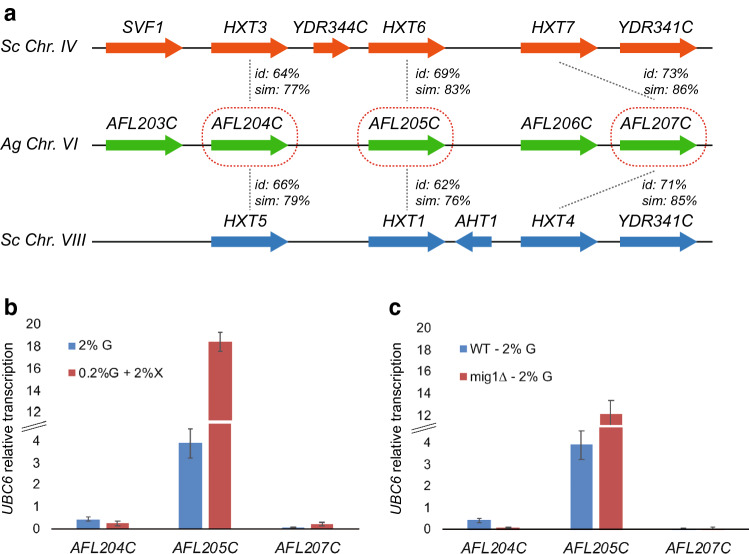
*HXT* homologs in *A. gossypii.* **a** Comparison of the genomic region of the *A. gossypii* chromosome VI and the *S. cerevisiae* chromosomes IV and VIII. The *AFL204C*, *AFL205C* and *AFL207C* are sintenologs of *S. cerevisiae HXT* duplicated genes. The percentage of amino acid identity and similarity between the homologs is indicated. **b** qPCR gene expression analysis of *AFL204C*, *AFL205C* and *AFL207C* in the wild-type strain of *A. gossypii* grown in MA2-rich media containing either 2% glucose or 0.2% glucose plus 2% xylose. **c** qPCR gene expression analysis *AFL204C*, *AFL205C* and *AFL207C* in the wild-type and mig1∆ strains of *A. gossypii*

Considering the proteins Hxt1-7 and Gal2 as the most prominent glucose transporters in *S. cerevisiae*, a genomic comparison with *A. gossypii* revealed that orthologs for Hxt2 and Gal2 are missing in *A. gossypii*. However, three syntenic homologs of the *S. cerevisiae HXT1*, *HXT3-7* are clustered with a conserved gene order in the chromosome VI of *A. gossypii*: *AFL204C*, *AFL205C* and *AFL207C* owning a 62–73% of amino acid identity with the *S. cerevisiae* homologs (Fig. 1a).

A transcriptional analysis of the three *HXT* homologs showed that the highest mRNA levels corresponded to the *AFL205C* gene and its expression was induced 4.7-fold in culture media containing 0.2% of glucose and 2% of xylose (Fig. 1b). As previously mentioned, Mig1 is involved in the CCR mechanism in *S. cerevisiae*. Accordingly, our results showed that the expression of *AFL205C* was threefold higher (12.05 ± 1.31 *vs.* 3.89 ± 0.66) in a *mig1∆* mutant than in the wild-type strain (Fig. 1c), which indicates a possible *MIG1-*dependent regulatory mechanism in response to glucose levels, as previously described in *S. cerevisiae* [5]. The Mig1 regulator may be responsible of certain level of transcriptional repression of *AFL205C*, which is abolished in the *mig1∆* mutant (Fig. 1c). On the other hand, both *AFL204C* and *AFL207C* showed much lower expression than *AFL205C* (Fig. 1b-c). The expression of *AFL205C* in the GXX strain, which is able to grow using xylose as the only carbon source [4], was 4.4-fold lower when xylose was used as the only carbon source, suggesting that the expression of *AFL205C* is not induced (or partially repressed) when glucose is absent in the culture media. Thus, the expression of *AFL205C*, which is induced by low glucose levels and partially repressed both by high glucose concentrations and in the absence of glucose, resembles the regulation of *HXT2* in *S. cerevisiae* [22].

### Contribution of the *HXT* homologs to the xylose uptake in *A. gossypii*

**Fig. 2:**
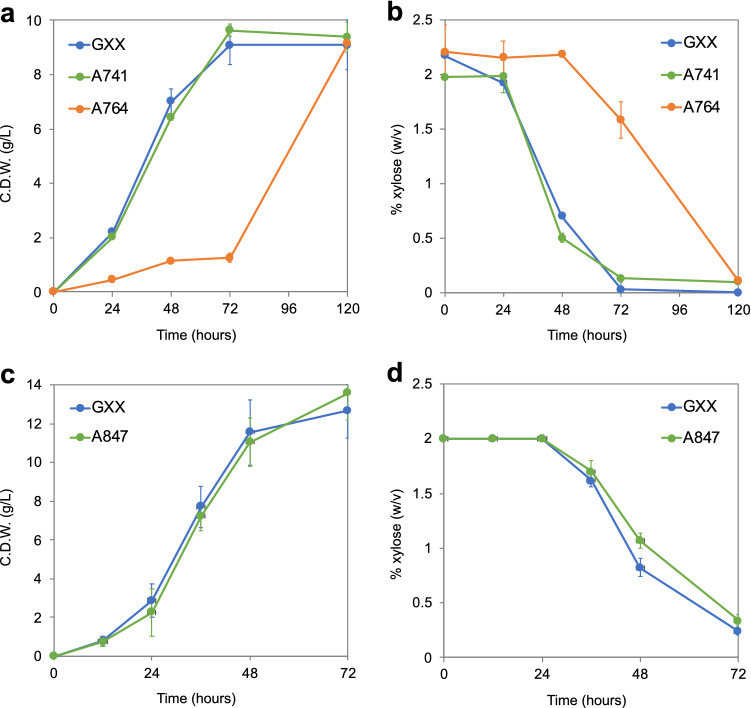
Deletion and overexpression of *HXT* homologs in *A. gossypii*. Biomass production (**a**) and xylose consumption (**b**) of the strains GXX, A741 (*afl205c∆*) and A764 (*afl204c∆*-*afl205c∆*-*afl207c∆*) grown in MA2 medium with 2% xylose as the only carbon source. Biomass production (**c**) and xylose consumption (**d**) of the strains GXX and A847 (GXX, *P*_*GP1D*_*-AFL205, P*_*GPD1*_*-AFL207C, P*_*GPD1*_*-AFL204C*) grown in MA2 medium with equal concentration (2% w/v) of glucose and xylose. The results are the average of three independent experiments. The error bars represent the standard deviations

The differences found on the expression profiles of the *HXT* homologs in *A. gossypii* can be associated with a different contribution of each gene to the glucose and xylose uptake. Consequently, single, double and triple gene deletions of the *AFL204C*, *AFL205C* and *AFL207C* genes were carried out in the parental strain GXX. Growth curves were obtained from xylose-based cultures, showing that single and double gene knock-outs of the *AFL204C*, *AFL205C* and *AFL207C* genes did not affect significantly the ability of *A. gossypii* to use xylose (Fig. 2a). In contrast, the growth of the triple mutant (A764 strain, *afl204c∆*-*afl205c∆*-*afl207c∆*) (Table 1) from xylose-based media was significantly delayed (Fig. 2a); however, the strain A764 was able to reach the same biomass as the GXX strain after 120 h. In good agreement, the consumption of xylose was also lagged in the strain A764, thus generating the growth defect in the triple mutant (Fig. 2b).

Compensatory mechanisms between the *AFL204C*, *AFL205C* and *AFL207C* genes must exist to counteract the absence of some of the *HXT* homologs. Furthermore, the ability of the triple mutant A764 to use xylose points out the existence of additional mechanisms for sugar transport in *A. gossypii* that enable the uptake of xylose when the three *HXT* homologs are inactivated. In this regard, two additional genes (*AFR602W* and *ADR091W*) were identified as potential candidates to encode sugar transporters owning a 30–40% of identity at protein level with the Hxt homologs*.*

### Overexpression of endogenous sugar transporters in *A. gossypii*.

Transcriptional regulatory mechanisms, which can repress the expression of hexose transporters, have been described [5]. A constitutive strong promoter from the *A. gossypii GPD1* gene was used to deregulate the transcription of the three *HXT* homologs. An engineered strain that overexpressed the three sugar transporters was obtained (strain A847) (Table 1). The growth ability of this strain was analyzed in cultures with equal concentration (2% w/v) of glucose and xylose to assess the utilization of both sugars. Our results showed that the simultaneous overexpression of the genes *AFL204C*, *AFL205C* and *AFL207C* (strain A847) did not improve the ability of *A. gossypii* to use xylose in the presence of glucose (Fig. 2c-d), thus indicating that transcriptional regulation is not the only mechanism involved in sugar selectivity in *A. gossypii*.

**Fig. 3:**
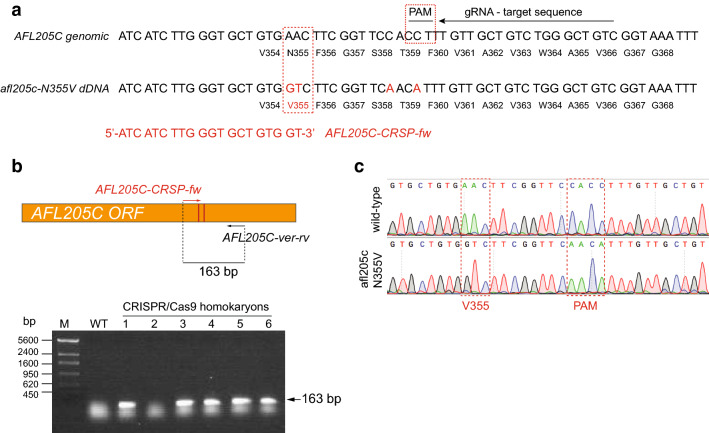
CRISPR/Cas9 edition of *AFL205C* in *A. gossypii*. **a** Representation of the genomic sequence of *AFL205C* corresponding to the amino acid N355. The protospacer adjacent motif (PAM) and the gRNA target sequence are indicated. The donor afl205c-N355V dDNA is shown below, where the nucleotide substitutions are indicated in red color. The nucleotide substitutions introduce the amino acid change (N355V) and eliminate the PAM sequence to avoid recurrent Cas9 activity. The sequence of the primer *AFL205C-CRSP-fw* used for analytical PCR is also shown in red color. **b** Strategy of the analytical PCR to confirm the presence of the genomic edition. A 163-bp amplicon is obtained when the edited sequence is present. PCR products from WT and 6 different homokaryotic clones were visualized by gel electrophoresis. **c** Chromatograms of the wild-type *AFL205C* locus and the mutant *afl205c-N355V* from the homokaryon 1 from B. The nucleotide changes are indicated

**Fig. 4:**
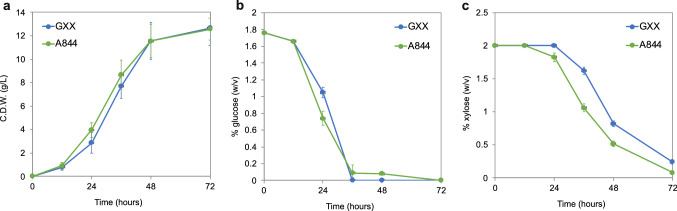
The amino acid replacement N355V in AFL205C improves the utilization of xylose in *A. gossypii*. Biomass production (**a**), glucose consumption (**b**) and xylose consumption (**c**) of the *A. gossypii* strains GXX and A844 (*P*_*GP1D*_-afl205c*(N355V), P_*GPD1*_*-AFL207C, P*_*GPD1*_*-AFL204C*) grown in MA2 medium with 2% xylose plus 2% glucose as the carbon sources. The results are the average of three independent experiments. The error bars represent the standard deviations

In this regard, it has been described that mutations in the residues that are important for sugar selectivity can enhance the affinity of the putative hexose transporters for xylose [10, 14, 23]. Hence, we decided to use a CRISPR/Cas9 system to introduce two point mutations into the *AFL205C* gene of the strain A847 (Fig. 3) that resulted in an amino acid change at residue 355 (N355V), which is conserved in the *A. gossypii* protein, and it has been involved in sugar specificity in *S. cerevisiae* [14]. The designed mutation in the strain A844 (Table 1) was confirmed by analytical PCR and DNA sequencing (Fig. 3). The A844 mutant strain showed a slight improvement of its growth performance in 2% glucose/xylose media (Fig. 4a); more importantly, the consumption of xylose in the presence of glucose by the A844 strain was significantly enhanced (Fig. 4b-c). Indeed, during the exponential phase, at 36 h of culture, the consumption of xylose was twofold higher in the A844 strain compared with the GXX strain (46% vs. 21% of xylose consumed) (Fig. 4c), thereby confirming the functionality of the replacement for the facilitation of the xylose uptake in the presence of glucose in *A. gossypii*.

Some strategies intended to improve the utilization of alternative sugars have described the overexpression of native hexose transporters, the overexpression of positive regulators or the inactivation of negative regulators [24–26]. In *A. gossypii*, the simultaneous overexpression of the three putative sugar transporters did not improve the co-utilization of glucose and xylose, therefore indicating that enzymatic regulatory mechanisms may hinder the utilization of xylose in the presence of glucose. Indeed, the amino acid substitution N355V in the AFL205C protein, which was previously related to the co-utilization of xylose and glucose in *S. cerevisiae* [10, 14], has been demonstrated to have a positive effect in the utilization of xylose in the presence of glucose in *A. gossypii*. Accordingly, the use of the *afl205c-N355V* allele will further promote the utilization of mixed sugars by engineered strains of *A. gossypii*.

## Electronic supplementary material

Below is the link to the electronic supplementary material.

Supplementary file1 (DOCX 21 kb)

## References

[1] Revuelta JL, Ledesma-Amaro R, Lozano-Martinez P (2017). Bioproduction of riboflavin: a bright yellow history. J Ind Microbiol Biotechnol.

[2] Lozano-Martínez P, Buey RM, Ledesma-Amaro R (2017). Engineering *Ashbya gossypii* strains for de novo lipid production using industrial by-products. Microb Biotechnol.

[3] Díaz-Fernández D, Aguiar TQ, Martín VI (2019). Microbial lipids from industrial wastes using xylose-utilizing *Ashbya gossypii* strains. Bioresour Technol.

[4] Díaz-Fernández D, Lozano-Martínez P, Buey RM (2017). Utilization of xylose by engineered strains of *Ashbya gossypii* for the production of microbial oils. Biotechnol Biofuels.

[5] Gancedo JM (1998). Yeast carbon catabolite repression. Microbiol Mol Biol Rev.

[6] Leandro MJ, Fonseca C, Gonçalves P (2009). Hexose and pentose transport in ascomycetous yeasts: an overview. FEMS Yeast Res.

[7] Jeffries TW, Jin Y-S (2004). Metabolic engineering for improved fermentation of pentoses by yeasts. Appl Microbiol Biotechnol.

[8] Kwak S, Jin Y-S (2017). Production of fuels and chemicals from xylose by engineered Saccharomyces cerevisiae: a review and perspective. Microb Cell Fact.

[9] Sharma NK, Behera S, Arora R (2018). Xylose transport in yeast for lignocellulosic ethanol production: current status. J Biosci Bioeng.

[10] Zhang GC, Liu JJ, Kong II (2015). Combining C6 and C5 sugar metabolism for enhancing microbial bioconversion. Curr Opin Chem Biol.

[11] Li H, Schmitz O, Alper HS (2016). Enabling glucose/xylose co-transport in yeast through the directed evolution of a sugar transporter. Appl Microbiol Biotechnol.

[12] Jeffries TW (2006). Engineering yeasts for xylose metabolism. Curr Opin Biotechnol.

[13] Subtil T, Boles E (2012). Competition between pentoses and glucose during uptake and catabolism in recombinant Saccharomyces cerevisiae. Biotechnol Biofuels.

[14] Farwick A, Bruder S, Schadeweg V (2014). Engineering of yeast hexose transporters to transport d-xylose without inhibition by d-glucose. Proc Natl Acad Sci U S A.

[15] Nijland JG, Shin HY, De Jong RM (2014). Engineering of an endogenous hexose transporter into a specific d-xylose transporter facilitates glucose-xylose co-consumption in Saccharomyces cerevisiae. Biotechnol Biofuels.

[16] Wang C, Bao X, Li Y (2015). Cloning and characterization of heterologous transporters in Saccharomyces cerevisiae and identification of important amino acids for xylose utilization. Metab Eng.

[17] Young E, Lee S, Alper H (2010). Optimizing pentose utilization in yeast: the need for novel tools and approaches. Biotechnol Biofuels.

[18] Jiménez A, Santos MA, Pompejus M, Revuelta JL (2005). Metabolic engineering of the purine pathway for riboflavin production in Ashbya gossypii. Appl Environ Microbiol.

[19] Aguiar TQ, Dinis C, Domingues L (2014). Cre-loxP-based system for removal and reuse of selection markers in *Ashbya gossypii* targeted engineering. Fungal Genet Biol.

[20] Mateos L, Jiménez A, Revuelta JL, Santos MA (2006). Purine biosynthesis, riboflavin production, and trophic-phase span are controlled by a Myb-related transcription factor in the fungus *Ashbya gossypii*. Appl Environ Microbiol.

[21] Jiménez A, Muñoz-Fernández G, Ledesma-Amaro R (2019). One-vector CRISPR/Cas9 genome engineering of the industrial fungus *Ashbya gossypii*. Microb Biotechnol.

[22] Ozcan S, Johnston M (1995). Three different regulatory mechanisms enable yeast hexose transporter (HXT) genes to be induced by different levels of glucose. Mol Cell Biol.

[23] Young EM, Tong A, Bui H (2014). Rewiring yeast sugar transporter preference through modifying a conserved protein motif. Proc Natl Acad Sci U S A.

[24] Ostergaard S, Olsson L, Johnston M, Nielsen J (2000). Increasing galactose consumption by Saccharomyces cerevisiae through metabolic engineering of the GAL gene regulatory network. Nat Biotechnol.

[25] Rossi G, Sauer M, Porro D, Branduardi P (2010). Effect of HXT 1 and HXT 7 hexose transporter overexpression on wild-type and lactic acid producing Saccharomyces cerevisiae cells. Microb Cell Fact.

[26] Lee KS, Hong ME, Jung SC (2011). Improved galactose fermentation of Saccharomyces cerevisiae through inverse metabolic engineering. Biotechnol Bioeng.

