# Supplementary material for: Sugar transport for enhanced xylose utilization in Ashbya gossypii
Source: J Ind Microbiol Biotechnol. 2020 Oct 9;47(12):1173–9. doi: 10.1007/s10295-020-02320-5 (PMC7728639; doi:10.1007/s10295-020-02320-5)
Supplement: Supplementary file 1 — Supplementary file1 (DOCX 21 kb) [file 10295_2020_2320_MOESM1_ESM.docx]

**Sugar transport for enhanced xylose utilization in *Ashbya gossypii***

David Díaz-Fernández, Gloria Muñoz-Fernández, Victoria Isabel Martín, José Luis Revuelta and Alberto Jiménez

Metabolic Engineering Group, Departamento de Microbiología y Genética, Universidad de Salamanca, Campus Miguel de Unamuno, E-37007 Salamanca, Spain.

**Corresponding author:** E-mail address: [alji@usal.es](mailto:alji@usal.es)

**Electronic Supplementary Material: Table S1**

**Table S1. List of primers used in this study.**

| ***Primer*** | ***Sequence*** | ***Purpose*** |
| --- | --- | --- |
| *AFL204C∆*-fw | 5’-ATG ACA AGT ATC GTT ATC ACC TCA CCC ATT TCA GGT AGA CCG ACT ATT TGA GAC TGC TGT GGG AAA AAC ACG TTC AAA TGC GGA TCC CCG GGT TAA TTA A-3’ | *AFL204C* deletion |
| *AFL204C∆*-rv | 5’-GAT ATG CAA GCG GTT GAC CCA GCA GCT GAC GGT CAC CCC ACA CCT CTG CAT ACC TTT CTG CAC AAG GCA TGC AGT TAG AAT TCG AGC TCG TTT AAA C-3’ | *AFL204C* deletion |
| *AFL205C∆*-fw | 5’-GTT ATA ATA AAC CAT CGT TCA GAA TAT TTG CCG TTA AGA CTT CCA TTG AGG AAT AAT AAC TAA TAC TTG TCG GTA CAA TGC GGA TCC CCG GGT TAA TTA A-3’ | *AFL205C* deletion |
| *AFL205C∆*-rv | 5’-AAT ATT AAT GAA CAT AGA TTA GGT TTA GGT TTT AAC CCG ATG ATT AGC AGA ACA AAA AAG TTT GTA GTT TAA AGT TTT TAG AAT TCG AGC TCG TTT AAA C-3’ | *AFL205C* deletion |
| *AFL207C∆*-fw | 5’-CCA ACT GTT CAC CGG TCA TCA GTT TGC AGT GTC GAG GAA ACT GTT TCT ATC ATA TTT TAG ACG CAG TCG CCT TCT CAA TGC GGA TCC CCG GGT TAA TTA A-3’ | *AFL207C* deletion |
| *AFL207C∆*-rv | 5’-GAA CAG ACC GTC TGC TAC TAG GCC GAA AGG GTA AAG CAG TTG TCA GTC AGT ACT TGC TGT TGC TTA TGG AAT GCC TGT CAG AAT TCG AGC TCG TTT AAA C-3’ | *AFL207C* deletion |
| loxPMK-*P_GPD1_*-*AFL204C*-fw | 5’-TTC GTA AAA TAA TCA AAT TTT TAT AAA AGT CGA AGA CGA CCG TTG GTC TCG TAA ATG ACA AGT ATC GTT ATC ACC TCA CGG ATC CCC GGG TTA ATT AA-3’ | *AFL204C* overexpression |
| loxPMK-*P_GPD1_*-*AFL204C*-rv | 5’-TTG CTC TCA ATG GGA TGC GAA TTC GAA CCA TCT CCC GAG AGC TCG CTT TTA GAC GGC GTC ATA CCT TCA GAA ACG GTC ATT GTG CGG TGT GTA TGT GTG G-3’ | *AFL204C* overexpression |
| loxPMK-*P_GPD1_*-*AFL205C*-fw | 5’-AAG AGT ATA TAA GGA CAG CCT CGA CAC GCT TTC GTA GAT CTG ATA TGT TTA ATG TTA TAA TAA ACC ATC GTT CAG AAT ATC GGA TCC CCG GGT TAA TTA A-3’ | *AFL205C* overexpression |
| loxPMK-*P_GPD1_*-*AFL205C*-rv | 5’-TTC GAA GGC TTG TTC GAT GCT GTA GAC GAA TGT GTG CTC ATC ACG GAG CGG TTG TCA GGT TGT GCA ATT GCT GCA GAC ATT GTG CGG TGT GTA TGT GTG G-3’ | *AFL205C* overexpression |
| loxPMK-*P_GPD1_*-*AFL207C*-fw | 5’-AAC CTA ACA ACA TCT ACA GTA TAT AAG GAT AGA TGA GAT AGA TTA ACC CTC TTT CCA ACT GTT CAC CGG TCA TCA GTT CGG ATC CCC GGG TTA ATT AA-3’ | *AFL207C* overexpression |
| loxPMK-*P_GPD1_*-*AFL207C*-rv | 5’-CAA ATA CTT CTT TTG CGA TGC CGC GGA TAC CTC TGT ACC CAC GGA CCG CAT ATC ACT GTG TAA AAC CTG ACG GCT CAT TGT GCG GTG TGT ATG TGT GG-3’ | *AFL207C* overexpression |
| gAFL205C-N355V-fw | 5’-TGC TGT GTT CTT CGG TTC AAC ATT TGT TGC-3’ | *Target gene AFL205C CRISPR-CAS9* |
| gAFL205C-N355V-rv | 5’-CCG AAG AAC ACA GCA CCC AAG ATG ATC G-3’ | *Target gene AFL205C CRISPR-CAS9* |
| AFL205C-CRSP-fw | 5’-TCG TTC CAG AGT CTC CTC GT-3’ | *Analytical PCR* |
| AFL205C-ver-rv | 5’-CCC CGT TAG GCC ACA ACT TA-3’ | *Analytical PCR* |
| AFL204C-a | 5’-GTA TCG GCC CCT CAC TTA GC-3’ | *Analytical PCR* |
| AFL205C-a | 5’-CGT GAT GGC AGT ATA GCG GT-3’ | *Analytical PCR* |
| AFL207C-a | 5’-GCT TCT GTG TTC CCT CGT GA-3’ | *Analytical PCR* |
| KanB1 | 5’-CTG CAG CGA GGA GCC GTA AT-3’ | *Analytical PCR* |
| *AFL204C-fw* | 5’-CTA CCC AAG GCC CGG ATG TCT AT-3’ | *AFL204C*  qRT-PCR |
| *AFL204C-rv* | 5’-TCT GCC AAA TTT GTC AAC GGT GTA-3’ | *AFL204C*  qRT-PCR |
| *AFL205C-fw* | 5’-CAG CGT GTT ATT ATG GGT GTT ATG-3’ | *AFL205C*  qRT-PCR |
| *AFL205C-rv* | 5’-AGT CGC GAA GCA GAA AAT GTA GAT-3’ | *AFL205C*  qRT-PCR |
| *AFL207C-fw* | 5’-GGG CCC CAA TTG CGT ATG TCA-3’ | *AFL207C*  qRT-PCR |
| *AFL207C-rv* | 5’-TAT GCC GGC AGC TTG TTT GTC ACT-3’ | *AFL207C*  qRT-PCR |
| *UBC6-fw* | 5’-CAA CGA TAC TGA CTG GGC TGC TAA-3’ | *UBC6*  qRT-PCR |
| *UBC6-rv* | 5’-GGC GCG TAT CCT ATC CTC TGG-3’ | *UBC6*  qRT-PCR |
